# Immediate lymph node extraction improves retrieval rate following axillary lymph node dissection: an effective approach to improving guideline-concordant breast cancer care in Nigeria

**DOI:** 10.3332/ecancer.2023.1609

**Published:** 2023-10-02

**Authors:** Olalekan Olasehinde, Matteo Di Bernardo, Akinwumi Oluwole Komolafe, Oluwatosin Zainab Omoyiola, Funmilola Olanike Wuraola, Omolade Betiku, Opeyemi Ogunrinde, Adewale Aderounmu, Olaejirinde Olaniyi Olaofe, Adeyemi Adefidipe, Ese Ewoye, Tajudeen Olakunle Mohammed, Fisayo Oyeneye, Adewale Oluseye Adisa, Olusegun Isaac Alatise, Ganiyat Omoniyi-Esan

**Affiliations:** 1 Department of Surgery, Obafemi Awolowo University, Ile-Ife 220282, Nigeria; 2 African Research Group for Oncology, Obafemi Awolowo University, Ile-Ife 220282, Nigeria; 3 Department of Morbid Anatomy and Forensic Medicine, Obafemi Awolowo University Teaching Hospitals Complex, Ile-Ife 220282, Nigeria

**Keywords:** breast, cancer, lymph-node, axillary dissection

## Abstract

**Background:**

Axillary lymph node staging is essential for making therapeutic decisions and for prognostication. A minimum of ten lymph nodes is recommended for accurate staging. This study describes the process and outcomes of an audit cycle that resulted in a novel intervention instituted to improve concordance with guidelines.

**Methods:**

The study began with a retrospective audit of lymph node retrieval following axillary lymph node dissection (ALND). Subsequent phases evaluated the efficacy of immediate lymph node extraction before fixation by comparing the mean number of lymph nodes and the proportion of guideline-concordant cases to retrospective data and concurrent cases without the intervention.

**Results:**

The mean number of lymph nodes retrieved in the retrospective phase was 5.2, which is less than the recommended threshold. The intervention resulted in a significant increase in lymph node retrieval over the baseline rate (13.7 versus 5.2, *p* = 0.026). There was also a significantly higher number of lymph nodes following the intervention compared to concurrent cases managed during the same period without the intervention (13.7 versus 7.9, *p* = 0.004). The concordance rate was 89% in the intervention group compared to 47% in the non-intervention group (*p* = 0.019). There was no significant difference when the intervention was administered by either surgeons or pathologists (13.5 versus 12, *p* = 0.25).

**Conclusion:**

Immediate extraction of lymph nodes resulted in significant improvement of concordant lymph node retrieval in all phases of the study. We recommend that this practice be validated in larger cohorts for possible recommendation as an effective way of improving lymph node retrieval following ALND.

## Introduction

Axillary lymph node staging plays an important role in the management of breast cancer. It is essential for making correct therapeutic decisions and for prognostication [1, 2]. Axillary lymph node involvement sometimes indicates the need for more aggressive surgical or adjuvant therapies [3]. As a prognostic marker, regional lymph node involvement correlates with important clinical outcomes such as recurrence and mortality [4]. Axillary lymph node staging is performed either by performing a sentinel lymph node biopsy or an axillary lymph node dissection (ALND) [5–7], the former being increasingly utilised in many clinical scenarios [8]. ALND, however, remains the more common staging modality in Nigeria and many other low- and middle-income countries (LMICs) due to late presentation and infrastructural limitations [9, 10].

Measures and guidelines to improve the accuracy of lymph node staging have been developed over the years. A higher number of examined nodes is often associated with an increased probability of finding tumour-positive nodes [11–13]. A minimum of ten lymph nodes is often recommended for proper staging of the axilla following ALND [14–16]. Removal or examination of fewer than recommended nodes might result in under-treatment and other erroneous clinical judgements. The number of nodes retrieved during ALND is considered one of the metrics for assessing the quality of breast cancer care. The surgeon is expected to perform adequate dissection to ensure a sufficient number of lymph nodes are removed, while the pathologist should carefully examine the specimen to extract all removed nodes for histological appraisal. To ensure adherence to stipulated practice guidelines, a periodic audit is essential.

In Nigeria, the majority of patients present with late-stage disease with axillary node involvement, often necessitating ALND [13]. The practice of ALND and staging has, however, not been thoroughly evaluated in the Nigerian context. Being the most commonly performed axillary procedure in Nigeria and in many LMICs, it is essential to evaluate the practice and determine if quality standards are met. In this study, we performed an audit of axillary nodal staging in a Nigerian hospital. We also evaluated the impact of a novel intervention that aimed to address the pitfalls observed during the audit. We present the steps adopted in the audit cycle, from initiation to implementation. We consider the results stemming from this audit cycle, a potentially generalisable solution to improve lymph node evaluation in breast cancer care.

## Methods

This study was carried out at Obafemi Awolowo University Teaching Hospital (OAUTH), a tertiary hospital in South West Nigeria. The study began with an audit of lymph node yield and staging following ALND. This resulted in the rigorous evaluation of an intervention that aimed to improve the accuracy of lymph node staging. The research occurred in five phases.

### Phase I: retrospective review

A retrospective review of available operative and pathology reports of patients who underwent mastectomy for breast cancer between 1 January 2016 and 31 August 2020 was carried out. This analysis aimed to assess the practice of nodal staging.

During the period reviewed in this phase, the practice at OAUTH was to fix surgical specimens in formalin immediately after removal in the operating room, and subsequently send samples to the pathology department for grossing and lymph node extraction at a later period. The timing of lymph node extraction often ranged between 24 and 36 hours after resection and formalin fixation of the specimen.

### Phase II: stake holders meeting

Following the results observed in phase I of the study, an intervention was proposed by surgeons and pathologists to measure the effect of an improved method for obtaining adequate lymph node yield. The intervention aimed at decreasing the inadequacies observed in phase I of the study. The intervention administered was based on the claim by surgeons that more nodes were being removed at surgery than were being reported by pathologists. Both surgeons and pathologists agreed to the intervention described in phase III of the study.

### Phase III: intervention (Immediate lymph node extraction from the resected specimen in the operating room before fixation) in a single surgical unit

This phase of the study was enacted for a year from 1 September 2020 to 31 August 2021. The main objective was to determine if the inadequacy observed in phase I was a result of inadequate surgical resection or a result of a challenge with the identification and counting of the lymph nodes (the ‘cut’ or the ‘count’). An intervention was designed, which entailed that surgeons extract lymph nodes into separate vials in the operating room, immediately after en-bloc resection and before fixation in formalin.

The intervention was adopted in only one of the two surgical units involved in breast cancer management while the other maintained routine practice. While both units could have administered the intervention and results compared to the retrospective data, there was a need to eliminate the confounding effect of the evolving and improving landscape of practices among surgeons and pathologists which could account for the differences observed. Comparisons were, therefore, made between the two surgical units based on the cases operated concurrently during this phase of the study to control for time trend effects.

### Phase IV: immediate lymph node extraction by both surgical units

The fourth phase of the study was implemented over 7 months, from 1 September 2021 to 30 March 2022. During this period, both surgical units implemented the intervention. This fourth phase also enabled studying the differences before and after the intervention separately for both surgical units, to ensure that there were no biases in the effect of the intervention based on the competencies of the surgeons in the two surgical units.

### Phase V: introduction of immediate lymph node extraction into routine practice

Following phase IV, a stakeholders meeting was reconvened where the findings from the intervention were discussed by surgeons and pathologists. A final validation step was implemented as phase V to apply the learned results into routine practice. Fresh mastectomy specimens were transferred to the pathology department for immediate lymph node retrieval by pathologists before fixation, to complete the process within an hour of specimen removal. Pathologists were informed at least 24 hours before an upcoming mastectomy. Findings in this phase were compared with that of previous phases.

### Data collection and statistical analysis

Data were obtained from the OAUTH breast cancer REDCap database, pathology database, and theatre records.

All statistical analyses were done in R-studio 2022.12.0, using R 4.2.1. Statistical analyses and visualisations were constructed using ggplot2, ggBrackets, and ggpubr packages. The number of lymph node retrievals between groups was compared using the two-sided student’s *T*-test, while comparisons of proportions of concordant resections (≥10 lymph nodes) were done using the two-sided two-proportion *Z*-test. Boxplots depict the first to –third Inter quartile ranges (IQRs), the intermediate line represents the median, and the whiskers represent 1.5*IQR above and below the first and third IQRs, while barplots simply depict the proportions of concordant cases.

### Ethical considerations

This study has received the approval of the institutional ethical review committee.

## Results

A total of 214 mastectomies were reviewed across the study period. The surgeries split across the four phases: 114 (53%) in phase I, 39 (18%) in phase III, 26 (12%) in phase IV, and 35 (16%) in phase V. Of these, 159 (74%) of the operations were performed in the presence of a consultant surgeon, while 23 (11%) were performed by only residents, and in 32 cases (15%), the cadre of the surgeon could not be ascertained. Neoadjuvant chemotherapy (NAC) was administered in 118 cases (55%) while 40 (19%) did not receive this treatment (Table 1).

We evaluated the potential effect of NAC and the cadre of surgeons on the number of lymph nodes reported. Neither the administration of NAC nor the cadre of the surgeon affected the number of harvested nodes (*p*-value = 0.340 and 0.38, respectively). Other variables such as age, clinical stage and grade were also not significantly associated with the number of harvested nodes (*p* = 0.97, 0.26 and 0.79, respectively).

### Retrospective evaluation of lymph node retrieval

**Figure 1.**
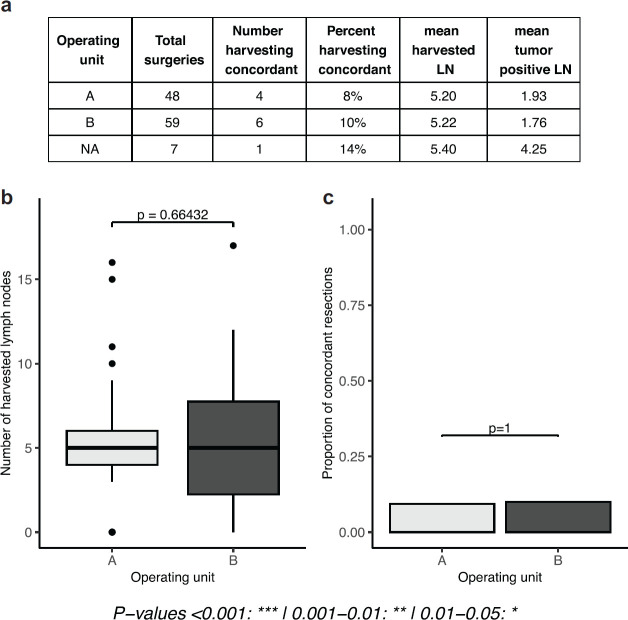
Evaluation of phase I results (number and proportion of concordant lymph nodes in a retrospective cohort). (a): tables of results, separated by operating unit. (b): boxplots depicting the number of lymph nodes extracted, separated by operating unit. (c): proportion of concordant lymph node extractions (separated by operating unit).

Findings in this phase of the study show a low level of guideline concordant axillary lymph node staging with only about 9% of cases overall reporting ≥10 lymph nodes. Mean harvested lymph nodes was found to be 5.20 and 5.22 for units A and B, respectively, and only about 8% and 10% of mastectomies had resections equal to or above the guideline recommendation. No statistically significant difference was found between units (Figure 1).

### Lymph node yield after selective administration of the intervention (comparison of lymph node yields between intervention and control groups)

**Figure 2.**
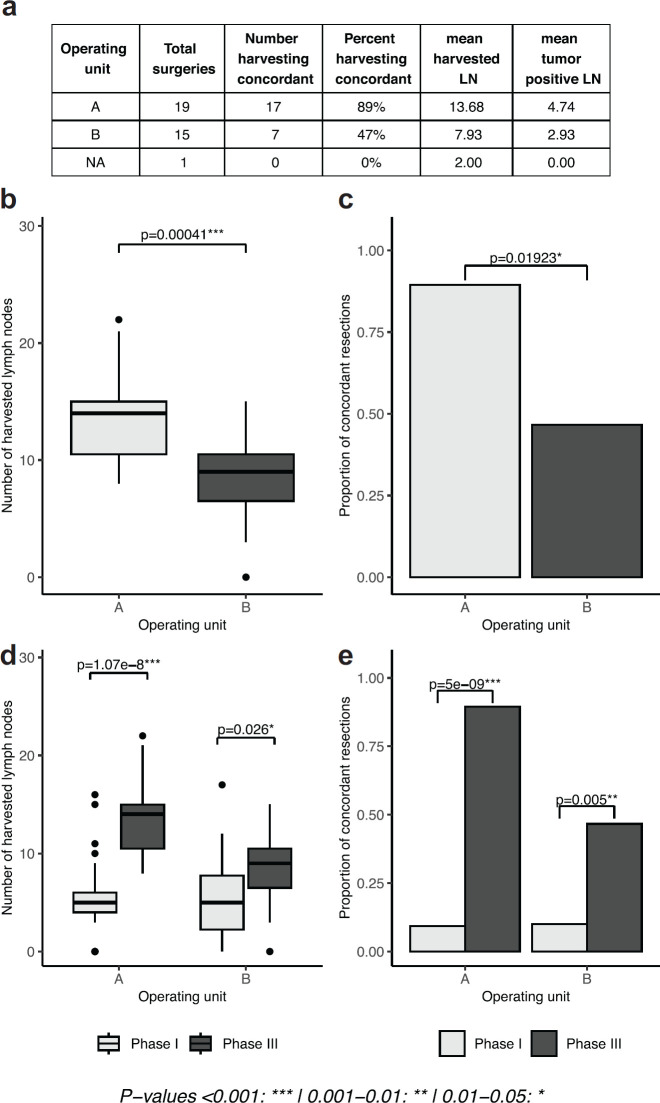
Lymph node yield after selective administration of intervention in one surgical unit (intervention versus control groups). (a): table of results, separated by operating unit. (b, d): boxplots depicting the number of lymph nodes extracted in phase I and phase I and III, respectively, separated by operating unit. (c, e): proportion of concordant lymph node extractions in phase I and phase I and III, respectively, separated by operating unit).

The results of the intervention showed significant improvement in the intervention unit. The mean number of harvested lymph nodes was significantly higher in unit A (which administered the intervention), compared to unit B (13.68 versus 7.93, *p* = 0.00041). The percentage concordance in unit A was almost double that of unit B which did not (89% versus 47%, *p*-value 0.01923). When the results of phases I and III were compared within each unit, the number of harvested lymph nodes (*p*-values 1.78e-8, 0.026, Figure 2) and the proportion of concordant cases (*p*-values 5e-8, 0.005, Figure 2) was significantly higher in phase III relative to phase I. The mean number of harvested lymph nodes, however, remained inadequate and concordance below 50% in the unit where the intervention was not executed.

### Lymph node yield following intervention in both surgical units

**Figure 3.**
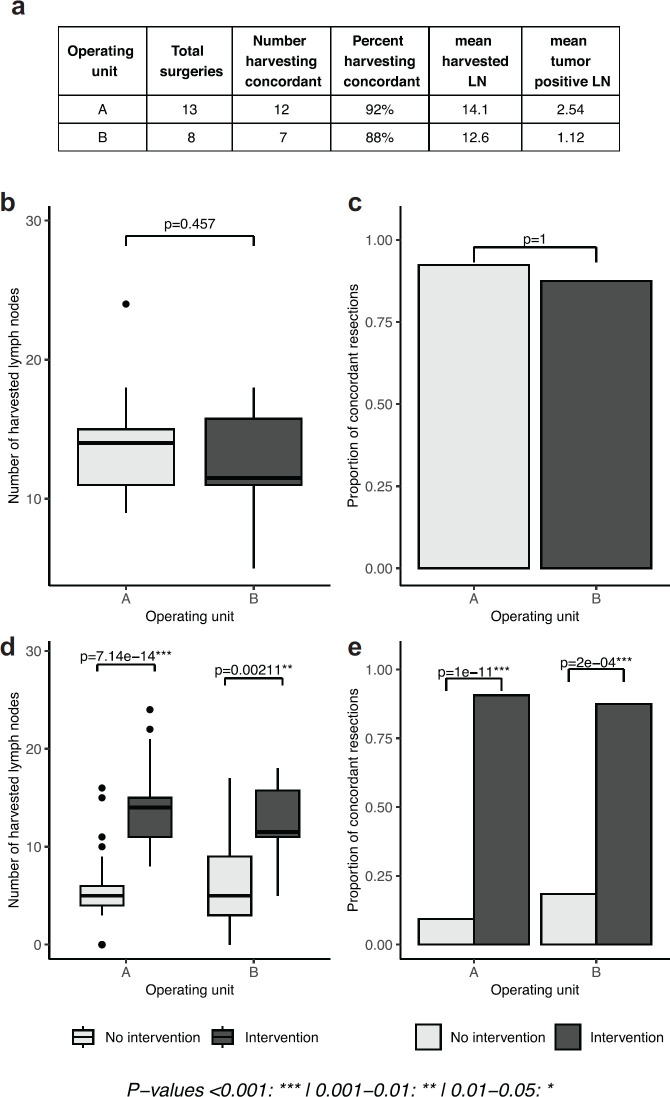
Lymph node yield following intervention in both surgical units. (a): tables of results, separated by operating unit. (b, d): boxplots depicting the number of lymph nodes extracted in phase IV and in cases with or without intervention, separated by operating unit. (c, e): proportion of concordant lymph node extractions in phase IV and in cases with and without intervention, separated by operating unit. No intervention period is defined as phase I for unit A, phase I and III for unit B; intervention period is defined as phase III and IV for unit A, phase IV for unit B).

Results stemming from phase IV, in which both units undertook the intervention, show that the positive results presented in phase III can be generalised across both units. The concordance rate was 92% in unit A and 88% in unit B (*p* = 1), with no statistical difference between units, a result that also translated to the mean number of harvested lymph nodes between the two units (14.1 in unit A and 12.6 in unit B, *p* = 0.457). The number of harvested lymph nodes, within units, was significantly higher in the intervention period (*p*-values 7.14e-14, 0.00211), a result recapitulated by the proportion of concordant cases (*p*-values 1e-11, 2e-4, Figure 3).

### Comparison of outcomes of intervention by pathologists with previous phases of the study

**Figure 4.**
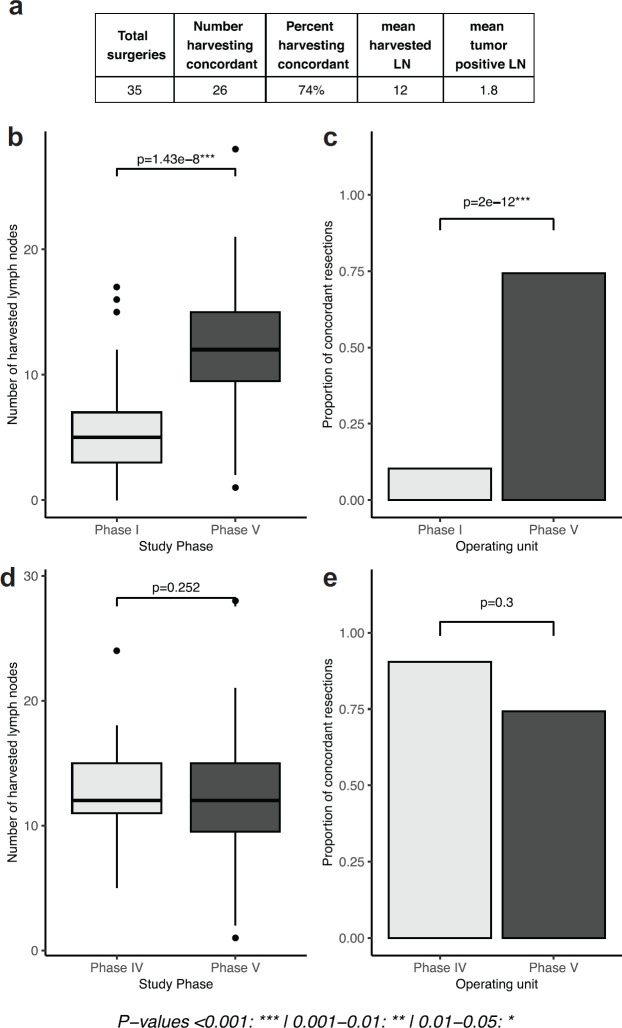
Comparison of intervention by pathologists with previous phases of the study. (a): tables of results, (b, d): boxplots depicting the number of lymph nodes extracted in phases I and V and phases IV and V, respectively, (c, e): proportion of concordant lymph node resections in in phases I and V and phases IV and V, respectively).

The mean number of harvested lymph nodes in this phase where pathologists undertook the intervention was 12, a significant improvement relative to baseline results in phase I (*p* = 1.43e-8). Findings in this phase were not significantly different from phase IV when the intervention was administered by surgeons (*p* = 0.252). Similar results were found when looking at the proportion of concordant cases, which was 74% in this phase. This was significantly higher than phase I (*p*-value 2e-12) and not significantly different from phase IV (*p* = 0.3, Figure 4).

### Correlation between the number of harvested nodes and the number of positive nodes

There was a positive correlation between the number of harvested lymph nodes and the number of tumour-positive nodes in the entire cohort (*R*^2^ = 0.09, *p* = 0.00002). When analysed based on the phase of the study, the correlation was significant only in phases I and III (*R*^2^ = 0.16, *p* = 0.0.00004 and *R*^2^ = 0.23, *p* = 0.003, respectively).

## Discussion

This project identified a gap in the practice of axillary nodal staging in a Nigerian hospital and proffered a novel solution which resulted in a significant leap from the baseline state. This study showed the significant impact of immediate lymph node extraction on lymph node retrieval rate. Our study showed that at all time points, immediate extraction of axillary lymph nodes after axillary dissection resulted in higher lymph node yield compared to the historical cohort and concurrent cases managed using the conventional approach. The intervention administered in this study resulted in a significant change from a 9% concordance rate in the retrospective phase and 47% in the prospective phase to a 74% concordance rate when pathologists extracted lymph nodes from the tissue immediately after removal.

Guidelines for the management of the axilla in patients with breast cancer recommend that a minimum of ten lymph nodes should be removed during ALND [14, 16]. The standard way to determine adherence to this practice guideline is by examining the report of the pathologist. This was the focus of phase I of the study which revealed a huge gap in practice about 90% non-concordance rate, with no significant differences between surgical units, cadre of surgeons, or receipt of NAC or otherwise as shown by some other studies [17–19]. In the prospective phase of the study, however, there was an improvement in the concordance rate even in the absence of an intervention with about 47% concordance rate in the unit which followed routine practice (unit B). This increase is a reflection of a more meticulous search by pathologists during surgery after having been made aware of the shortfalls in the retrospective phase. It might also have resulted from more meticulous dissection by the surgeons who were all aware of the study. Though better than baseline values, a 47% concordance rate is considered to be less than par. This finding is, however, similar to findings from a Nigerian study evaluating the impact of axillary lymph node involvement on recurrence which reported a 48.9% concordance rate [20].

The idea behind the intervention deployed in this study was based on a subjective observation that more nodes were being felt at surgery than were being reported as recovered by pathologists. The practice of immediate lymph node retrieval from fresh tissue specimens has not been widely described in literature and there is no data on this practice in breast surgery. Ours is one of the few studies to report on this practice. The effectiveness of the intervention is evident with the remarkable improvement in node retrieval rate after it was introduced to all surgical units.

In the final stage of the study, the intervention was shifted to the pathologists as a way of validating the intervention and introducing it into routine practice. This phase of the study showed that no statistical difference exists between surgeons or pathologists when carrying out this procedure of immediate lymph node extraction.

A potential concern with the intervention deployed in this study is the need to keep the cold ischemic time to the barest minimum to preserve the integrity of the tissues for histopathological and immunohistochemical analysis [21]. Certain processes including improved communication between departments, and the availability of a dedicated staff for the timely transportation of the specimen were put in place to ensure this. We recommend these logistical considerations as a part of the intervention to maintain quality tissue processing.

## Conclusion

Overall, we find that immediate lymph node extraction by the pathologist proved effective in increasing lymph node retrieval and guideline concordance following ALND. Although more data is required to confirm and generalise this result, we believe that this can be validated in other institutions.

## Conflicts of interest

The authors have no conflict of interest to declare.

## Funding

This study received no funding.

## Statement of ethics

The study was conducted in line with institutional ethical guidelines of the Obafemi Awolowo University Ile-Ife, Teaching Hospitals Complex, Ile-Ife, Nigeria. Ethical approval was obtained from the Ethics and Research Committee of the hospital.

## Author contributions

Olalekan Olasehinde conceptualised the study, Olalekan Olasehinde, Matteo Di Bernardo, Akinwumi Komolafe, Oluwatosin Zainab Omoyiola, Funmilola Olanike Wuraola, Adewale Aderounmu, Omolade Betiku, Olaojerinde Olaniyi Olaofe, Adewale Oluseye Adisa, Ganiyat Omoniyi-Esan contributed to the design of the study. Opeyemi Ogunrinde, Ese Ewoye, Fisayo Oyeneye, Adewale Adisa, Olusegun Isaac Alatise and Olalekan Olasehinde participated in data collection. Data analysis was done by Matteo Di Bernardo, while Olalekan Olasehinde, Akinwumi Oluwole Komolafe, Oluwatosin Zainab Omoyiola, Funmilola Olanike Wuraola, Omolade Betiku, Olaojerinde Olaniyi Olaofe, Adewale Aderounmu, Opeyemi Ogunrinde, Ese Ewoye, Adewale Oluseye Adisa, Olusegun Isaac Alatise, Tajudeen Olakunle Mohammed, Ganiyat Omoniyi-Esan contributed to data interpretation and writing of the manuscript. All authors approved the final version of the manuscript before submission.

## Data availability statement

The data used and analysed during the current study are available from the corresponding author on reasonable request.

## Figures and Tables

**Table 1.** Descriptive statistics.

	*n*	%
**Evaluated mastectomies (*n* = 214)**		
Study phase		
I	114	53
III	39	18
IV	26	12
V	35	16
Cadre of surgeon		
Consultant	159	74
Resident	23	11
Not stated	32	15
NAC received		
Yes	118	55
No	40	19
Unknown	56	26
Margin status (noted by pathologist)		
Positive	56	26
Negative	139	65
Not stated	19	9
Nottingham grade		
1	2	1
2	55	26
3	44	21
Not stated	90	42
No residual tumour	23	11
Histopathological diagnosis		
Invasive ductal carcinoma	111	52
Infiltrating ductal carcinoma	25	12
Ductal carcinoma (other)	7	3
Other carcinoma	12	6
Other malignant tumour	3	1
Remaining lymph metastasis only	7	3
Inflitrating adenocarcinoma	2	1
Mucinous adenocarcinoma	5	2
No residual tumour	37	17
Others	5	2
